# Ventriculoperitoneal (VP) Shunt-Associated Calcified Chronic Subdural Hematoma: Armored Brain Management With Shunt Revision

**DOI:** 10.7759/cureus.103255

**Published:** 2026-02-09

**Authors:** Hajar Hamadi, Oumaima Monadi, Yassine Ait M'Barek, Lamia Benantar, Khalid Aniba

**Affiliations:** 1 Neurological Surgery, Ibn Tofail Hospital, Mohammed VI University Hospital, Marrakech, MAR

**Keywords:** armored brain, calcified subdural hematoma, chronic subdural hematoma, ventriculoperitoneal shunt, vp shunt complication, vp shunt revision

## Abstract

Calcified chronic subdural hematoma (CCSDH) or "armored brain" is a rare, late-stage complication that has been reported more often in children and young adults and may arise after ventriculoperitoneal (VP) shunting. We present a case of a 14-year-old male patient with cerebral palsy and a VP shunt presenting with intermittent headache. A non-contrast CT scan was consistent with bilateral CCSDH. The patient underwent shunt revision and remained asymptomatic during a follow-up period of two years. Through this case, we illustrate the importance of individualized management guided by clinical and imaging findings.

## Introduction

Calcified chronic subdural hematoma (CCSDH), also referred to as "armored brain," is a rare and late complication of long-standing subdural collections [1-4]. The incidence of CCSDH among chronic subdural hematomas (CSDHs) remains uncertain, with reported rates ranging from 0.8% to 10% in various series [2,3,5]. Although CSDH usually affects the elderly population, cases of calcification and ossification are more frequently observed in children and young adults, often associated with underlying etiologies such as hydrocephalus and shunt placement [2,5].

The pathogenesis remains unclear and appears multifactorial. Proposed mechanisms include prolonged organization of neomembranes with deposition of dense collagen, repeated microhemorrhages or persistent subdural hygroma with stagnation of blood products, intramembranous vascular thrombosis, and local metabolic or circulatory factors that favor calcium deposition and eventual ossification [1,6]. In patients with cerebrospinal fluid (CSF) shunts, chronic overdrainage is a recognized factor for subdural collections and has been frequently implicated in the development of CCSDH [6]. This report details the clinical and radiological findings, discusses the role of shunt physiology in pathogenesis, and outlines the management strategy.

## Case presentation

A 14-year-old male patient with a notable past medical history of cerebral palsy and hydrocephalus managed with a medium-pressure ventriculoperitoneal (VP) shunt placed at two years of age presented to the emergency department with an isolated headache. The headache was moderate in intensity (corresponding to a 4/10 on the visual analog scale), intermittent over a period of seven days. He reported no other associated symptoms of vomiting, seizures, focal weakness, or alteration of consciousness. There was no history of recent head trauma, shunt infection, or dysfunction. On admission, the patient was stable. Neurological examination was normal with no papilledema on fundoscopic eye examination.

**Figure 1:**
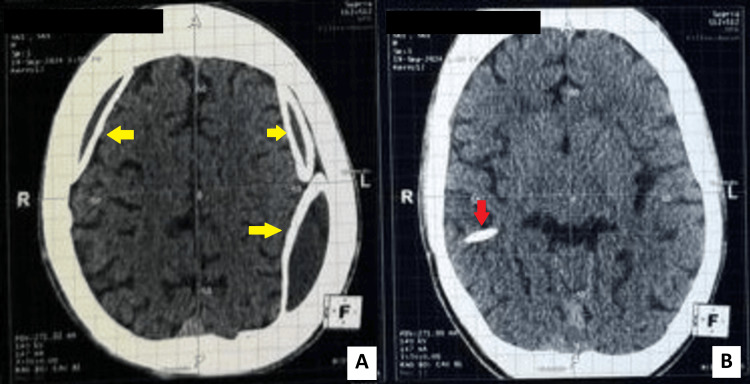
Preoperative axial non-contrast CT of the head. (A) CT scan of the head showing bilateral dense subdural laminar calcified collections encasing the convexity (yellow arrows). (B) CT scan of the head showing the VP shunt catheter projection toward the right lateral ventricle (red arrow) with a collapsed aspect of the lateral ventricle related to hyperdrainage. VP: ventriculoperitoneal

Non-contrast axial CT scan of the head showed bilateral crescentic extra-axial collections, the largest measuring 10 mm in thickness. Both collections were surrounded by dense laminar calcifications forming an image of a shell encasing both cerebral hemispheres. The calcified edges were continuous along the convexities on both sides. A VP shunt catheter was visible coursing toward the right lateral ventricle. CT scan of the head also showed no significant midline shift (Figure 1). Laboratory workup, including full blood count and coagulation profile, was within normal limits (Table 1). No signs of systemic infection were present.

**Table 1:** Preoperative laboratory blood test results. Patient's lab results showing normal values. INR: international normalized ratio; AST: aspartate aminotransferase; ALT: alanine aminotransferase

Test	Result	Reference Range
Hemoglobin	13.8 g/dL	12.0-16.0 g/dL
Hematocrit	41%	36-46%
White blood cell count	7.2 × 10⁹/L	4.0-11.0 × 10⁹/L
Platelet count	280 × 10⁹/L	150-450 × 10⁹/L
Prothrombin time	12.0 s	11.0-15.0 s
INR	1.0	0.9-1.2
Sodium	136 mmol/L	135-145 mmol/L
Potassium	4.2 mmol/L	3.5-5.0 mmol/L
Urea	12 mg/dL	7-20 mg/dL
Creatinine	0.6 mg/dL	0.4-0.9 mg/dL
Fasting glucose	95 mg/dL	70-140 mg/dL
AST	20 U/L	10-40 U/L
ALT	16 U/L	7-45 U/L
C-reactive protein	1.2 mg/L	<5 mg/L

**Figure 2:**
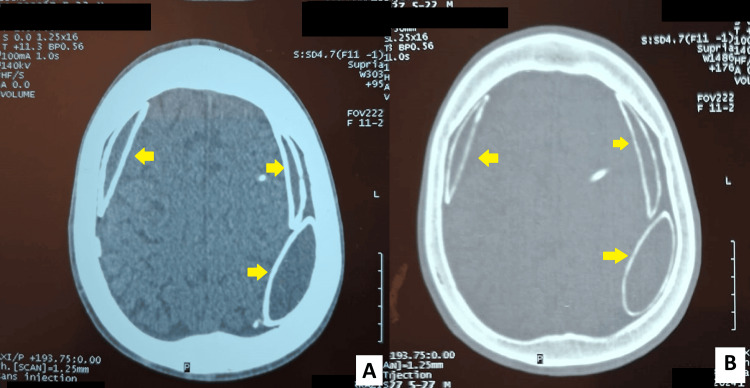
Control CT scan at two years follow-up showing a stable aspect of the collections. (A) Parenchymal window showing persistent hypodense organized calcified subdural collections (yellow arrows). (B) Bone window emphasizing dense laminar calcification along the convexities (yellow arrows).

Initial management was conservative and symptomatic with analgesia and hydration. Given the patient’s shunt dependency and the recognized association between chronic shunt overdrainage and subdural collections, we elected to perform shunt revision for pressure adjustment as the first therapeutic step. After revision, the patient remained neurologically stable and reported resolution of headaches within the hospital stay. He remained asymptomatic and was discharged home in good condition on the fourth postoperative day. Clinical follow-up visits were scheduled at one month, three months, six months, one year, and two years after shunt revision. At all visits, the patient remained asymptomatic with no headaches or neurological deficits. Furthermore, radiological control showed a stable aspect of the collections (Figure 2). No further surgical interventions were scheduled, given the stable clinical and radiological aspects.

## Discussion

"Armored brain" or "Matrioska head" - derived from the famous Russian doll - is a term used to describe bilateral CSDH with extensive calcifications or ossifications resulting in a "bone under bone" or "double skull" appearance on neuroimaging [2,6]. This entity is exceedingly rare, with the majority of cases reported as single observations diagnosed on CT in the literature [2]. Although the reported incidence varies across series, CCSDHs account for approximately 0.8% to 10% of CSDHs [3,6,7].

The mechanism underlying calcification is multifactorial and remains unclear [2,4,6]. Persistence of subdural fluid, repeated microhemorrhages within the membrane, and local vascular, inflammatory, and metabolic conditions that promote calcium deposition have all been implicated [1,2,6,8]. Reported etiologies include traumatic subdural hematoma, ventricular shunting procedure, post-meningitis or encephalitis, and tumors of the central nervous system [3,8]. In patients with VP shunts, chronic CSF overdrainage is a well-recognized cause of subdural hematoma and recurrent subdural bleeding and is considered a major contributor to the development of CCSDH [1,2,7]. These mechanisms help explain the higher prevalence of CCSDHs in pediatric and young adult populations [1,2,7].

The clinical manifestation of CCSDH is characterized by an insidious course with slow progression of neurological signs and symptoms. Many patients remain asymptomatic for a long period of time. When present, symptoms may include gait disturbance, chronic headache, visual disturbances, epileptic seizures, dysphasia, behavioral changes, memory impairment, paresis or plegia, and, in severe cases, altered consciousness [2,3,8].

The management of CCSDH should be individualized. In asymptomatic patients or those with mild symptoms and stable imaging findings, conservative management with clinical and radiological surveillance is recommended [2,3]. In shunted patients, valve adjustment or revision is described as a low-risk first step aimed at the correction of CSF overdrainage and may lead to symptom resolution without intracranial surgery [3]. When symptoms are progressive, mass effect is significant, or conservative measures fail, surgical evacuation is indicated [2].

This case reinforces the value of a stepwise, individualized management approach based on clinical and radiological findings. Our patient was young, presented with only mild intermittent headaches responsive to analgesics, and had a normal neurologic examination. Imaging showed bilateral calcified CSDHs without significant mass effect. Given these parameters, surgical evacuation was unlikely to provide additional benefits. Shunt revision alone resulted in rapid symptom relief and clinical stability over a two-year follow-up period. This outcome supports prioritizing shunt revision before considering intracranial surgery in selected patients and aligns with previously reported cases documenting a favorable outcome with shunt correction alone [2].

## Conclusions

Calcification of CSDH is an unusual complication of VP shunt placement and may lead to an "armored brain" syndrome. While surgical evacuation remains the cornerstone for symptomatic subdural hematomas, this case underscores the importance of addressing CSF dynamics in VP shunted patients with CCSDH. An individualized approach guided by clinical presentation and radiological findings may prevent the need for more invasive intracranial procedures in selected cases. Given the limited evidence base - mostly composed of case reports and small series - careful patient selection and shared decision-making remain paramount to achieve a favorable outcome in CCSDH.
